# Prognostic biomarkers and regulatory mechanisms associated with lysine β-hydroxybutyrylation modification in prostate cancer

**DOI:** 10.3389/fmolb.2026.1778536

**Published:** 2026-04-10

**Authors:** Yonghui Meng, Jinjun He, An Yan, Bangwei Che, Kaifa Tang, Tao Zhang

**Affiliations:** 1 Department of Urology, The First Affiliated Hospital of Guizhou University of Traditional Chinese Medicine, Guiyang, Guizhou, China; 2 Department of Urology, Beijing University of Chinese Medicine Shenzhen Hospital (Longgang), Shenzhen, Guangdong, China

**Keywords:** aurora kinase B, kinesin family member 4A, lysine β-hydroxybutyrylation, prostate cancer, TPX2 microtubule nucleation factor

## Abstract

**Background:**

Prostate cancer (PCa) is a highly prevalent malignant tumor in males. Lysine β-hydroxybutyrylation (Kbhb), an emerging post-translational modification, plays a critical role in tumorigenic processes. However, its functional mechanism in PCa remains elusive. This study aims to identify Kbhb-related prognostic genes in PCa and provide novel insights for its diagnosis, prognosis and treatment.

**Methods:**

Candidate genes were obtained through differential expression analysis and intersection analysis. Univariate Cox proportional hazards model and least absolute shrinkage and selection operator (LASSO) regression were employed to screen Kbhb-related prognostic genes, and a risk model was constructed and validated. The expression levels of Kbhb modification and related prognostic genes were validated using Western blotting (WB), real-time quantitative PCR (RT-qPCR) and immunohistochemistry (IHC). Additionally, clinical correlation analysis, nomogram development and validation, immune infiltration analysis, tumor mutation burden (TMB) analysis, gene set enrichment analysis (GSEA), drug sensitivity prediction, molecular regulatory network analysis, drug targeting analysis, and molecular docking were performed.

**Results:**

Three genes--kinesin family member 4A (KIF4A), TPX2 microtubule nucleation factor (TPX2), and aurora kinase B (AURKB)—were identified as Kbhb-related prognostic genes. Results of WB indicated that the butyrylation level of the H3K27 (H3K27-Bu) protein was significantly upregulated in PCa tissues. RT-qPCR and IHC results demonstrated that the expression levels of KIF4A, TPX2, and AURKB were significantly higher in the PCa than normal tissues. A risk model based on these genes demonstrated discriminatory ability (AUC >0.6) and served as an independent prognostic factor alongside prostate-specific antigen (PSA). The prognostic nomogram showed high accuracy (AUC 0.82–0.88). High-risk patients exhibited distinct immune infiltration profiles and higher mutation frequencies in tumor protein 53 (TP53), Titin (TTN), and speckle-type POZ protein (SPOP). Drug sensitivity analysis linked the risk score to 24 compounds, while molecular docking suggested that estradiol and bisphenol A could target the identified hub genes.

**Conclusion:**

This study identified KIF4A, TPX2, and AURKB as reliable prognostic biomarkers for PCa. These findings provide a theoretical basis for understanding Kbhb-mediated mechanisms in PCa and offer novel targets for early diagnosis and precision therapy.

## 1 Introduction

Prostate cancer (PCa) is a malignant tumor originating from the epithelial cells of the prostate and represents one of the most common malignancies of the male genitourinary system (Zhou et al., 2024). Globally, its incidence ranks as the second-highest among male cancers, particularly prevalent in middle-aged and elderly men over 50 years of age, with the risk increasing significantly with age (MacKintosh et al., 2016; Down et al., 2024). Epidemiologically, approximately one in eight men may be diagnosed with PCa during their lifetime. While a subset of patients may progress to aggressive disease, the majority can achieve long-term survival through advanced and precise diagnosis approaches and standardized management (Halstuch et al., 2020; Sundaresan et al., 2025). The pathogenesis of PCa is closely linked to multiple factors, with core contributing factors including genetic susceptibility, sustained androgen stimulation, obesity and metabolic disorders, and chronic inflammation (Haghighi et al., 2023; Pang et al., 2025; Pérez-Gómez et al., 2023). In the early stages of PCa, specific symptoms are often absent. However, as the tumor enlarges and compresses the urethra or invades surrounding tissues, patients may experience urinary frequency, urgency, dysuria, weak or interrupted urine flow, and hematuria (Pérez-Gómez et al., 2023), severely impacting patients’ quality of life (Guan et al., 2020). It is therefore imperative to elucidate the molecular mechanisms driving PCa pathogenesis and progression, as this is fundamental to optimizing early detection, personalizing therapeutic strategies, and enhancing survival rates.

Lysine β-hydroxybutyrylation (Kbhb) is a novel post-translational modification that occurs on lysine residues of proteins, in which a β-hydroxybutyryl group is covalently attached to lysine residues via an amide bond, a reaction catalyzed by specific acyltransferases. Kbhb modification is conserved across both prokaryotes and eukaryotes (Chen C. et al., 2025). In contrast to conventional modifications such as acetylation and propionylation, Kbhb possesses a distinct chemical structure. Its modification sites are frequently located within functionally critical regions of proteins, where it can modulate molecular functions by altering protein conformation or interaction patterns (Ouyang et al., 2024). The dynamic equilibrium of Kbhb is maintained by the opposing actions of acyltransferases which may include homologs with potential functional variations and deacylases. Acyltransferases facilitate the transfer of the β-hydroxybutyryl acyl group from β-hydroxybutyryl-CoA to lysine residues (Xu et al., 2018), while deacylases catalyze the hydrolysis of the acyl group, thereby reversing the modification and ensuring its reversible regulation (Wei et al., 2017; Zhang et al., 2024). Kbhb represents a metabolically-regulated, reversible modification that participates in fundamental cellular processes, with its balanced state being crucial for cellular homeostasis. Firstly, it regulates gene expression by modifying histones, influencing chromatin accessibility, and subsequently activating or repressing the transcription of target genes (Xie et al., 2016). Secondly, it modulates the activity of metabolic enzymes, particularly those involved in glycolysis and the tricarboxylic acid (TCA) cycle, thereby optimizing cellular energy supply (Li et al., 2025). Thirdly, it enhances cellular stress adaptation; under conditions of nutrient deprivation or oxidative stress, Kbhb modifies stress-related proteins to promote cell survival (Tang et al., 2025). The equilibrium state of Kbhb is closely related to tumorigenesis. Emerging evidence indicates that β-hydroxybutyrate (βhb) directly suppresses mTOR signaling to curb tumor growth (Qin et al., 2024). In hepatocellular carcinoma, βhb triggers Kbhb modification of ALDOB at Lys108, disrupting its interaction with FBP and concurrently inhibiting mTOR and glycolysis. This pathway also restricts proliferation in renal, gastric, and liver cancers, highlighting a shared mechanism linking metabolic enzyme modification to mTOR control. This tumor-suppressive role of Kbhb is further supported by studies showing that elevated βhb levels, which drive Kbhb modification, are associated with suppressed tumor growth in other cancer models (Khoziainova et al., 2022). In PCa, disrupted Kbhb regulation may be closely associated with tumor initiation, progression, and drug resistance. Emerging evidence indicates that βhb, via Kbhb modification, can significantly inhibit the proliferation, migration, and invasion of PCa cells (Zhang and Li, 2024). These parallel cases highlight the potential of targeting Kbhb-regulated metabolic enzymes as a unified approach to disrupt distinct oncogenic pathways. Furthermore, given that metabolic reprogramming (including ketone body metabolism from which Kbhb is derived) can shape the tumor immune microenvironment (Guo et al., 2025), investigating the immunologic context of Kbhb-related signatures is warranted. Aberrant Kbhb regulation is likely to influence PCa pathogenesis through multiple mechanisms. Nonetheless, the precise mechanistic involvement of Kbhb in PCa, including validated targets and clinically actionable insights, remains poorly defined. Most studies focus on individual molecules (Zhu and Huang, 2025), lack direct links to clinical outcomes, and have no systematic prognostic model based on Kbhb-modification-related genes; their regulatory networks remain unclarified. Investigating the prognostic genes associated with both PCa and Kbhb holds promise for identifying novel biomarkers and potential therapeutic targets, thereby advancing precise diagnosis and personalized treatment strategies to improve patient outcomes.

**FIGURE 1:**
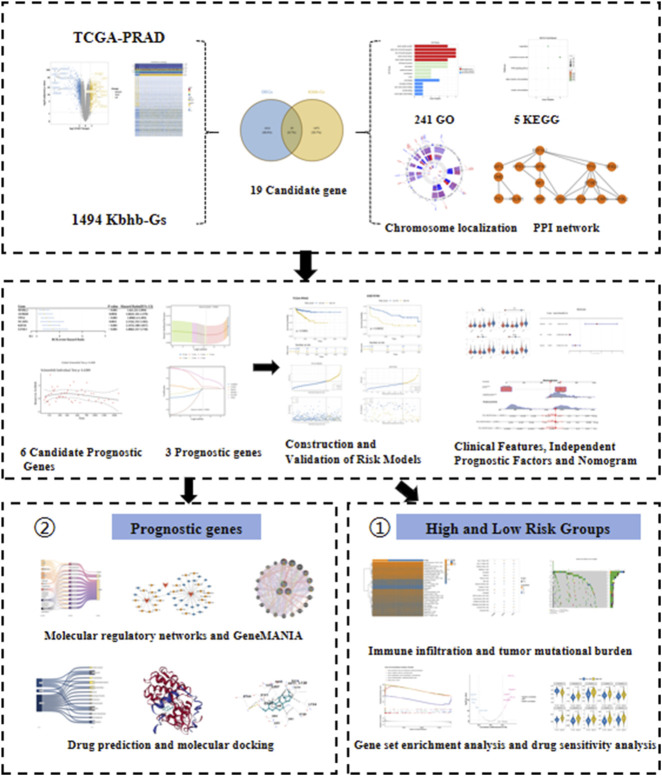
Analysis of the workflow.

Diverging from conventional prognostic markers, this study targets Kbhb modifications within a defined metabolism-epigenetics axis, with the aim to identify Kbhb-related prognostic genes in PCa and to construct a prognostic risk model. Analysis of the workflow is presented in Figure 1. Through subsequent analyses, including expression of prognostic biomarkers, development and evaluation of a nomogram, assessment of immune cell infiltration, functional enrichment analyses, and drug sensitivity profiling, we delineate the Kbhb-associated molecular roles underlying PCa pathogenesis and progression. Our findings hold promise for optimizing clinical management strategies and facilitating the development of novel therapeutics against PCa.

## 2 Materials and methods

### 2.1 Data collection

Transcriptomic profiles were retrieved from the Cancer Genome Atlas-Prostate Adenocarcinoma (TCGA-PRAD) database (https://xenabrowser.net/datapages/). A total of 483 PCa samples (tumor group) and 51 normal samples (normal group) from the TCGA-PRAD dataset were included in the training cohort, among which 408 PCa samples contained survival information (Supplementary Table S1). Additionally, 386 PCa samples with clinical data from the training cohort were used to construct a clinical feature model for further analysis. The GSE70769 dataset was downloaded from the Gene Expression Omnibus (GEO, https://www.ncbi.nlm.nih.gov/geo/) as the validation cohort. Among these, 92 PCa samples with complete survival information were included for the validation of the prognostic model. In addition, a total of 1,494 Kbhb genes were obtained from the reference (Hu et al., 2024).

### 2.2 Differential expression analysis

In the training cohort, the “DESeq2” package (v 1.38.0) (Love et al., 2014) was used to identify differentially expressed genes (DEGs) between the tumor group and the normal group, with screening criteria set at an adjusted p-value (*P. adj*) < 0.05 and |log2 fold change (FC)| > 1.5. The “ggplot2” package (v 3.4.4) was employed to generate a volcano plot of the DEGs, displaying the top 10 upregulated and downregulated genes based on |log2FC|. Additionally, the “ComplexHeatmap” package (v 2.14.0) (Gu et al., 2016) was utilized to construct a heatmap illustrating the expression levels of the top 100 upregulated and downregulated genes.

### 2.3 Functional enrichment and PPI network analysis

To further identify candidate genes, we performed an intersection analysis between differentially expressed genes (DEGs) and Kbhb genes using the “ggvenn” package (v 0.1.9). The resulting overlapping gene set was defined as candidate genes. To explore the potential signaling pathways associated with these candidate genes, Kyoto Encyclopedia of Genes and Genomes (KEGG) enrichment analyses were conducted using the “clusterProfiler” package (v 4.7.1.003). To investigate the chromosomal distribution of the candidate genes, their genomic locations were annotated and visualized using the “RCircos” package (v 1.2.2) (Zhang et al., 2013), which generated a circos plot illustrating their positions across chromosomes. Furthermore, to examine the functional interactions among the candidate genes at the protein level, the gene list was submitted to the Search Tool for the Retrieval of Interacting Genes/Proteins (STRING, http://string-db.org). A protein-protein interaction (PPI) network was constructed with an interaction score threshold set to >0.15.

### 2.4 Acquisition of prognostic genes

To identify prognostic genes, univariable Cox regression analysis and Least Absolute Shrinkage and Selection Operator (LASSO) regression analysis were performed on samples with complete survival data from the TCGA-PRAD training cohort. Univariable Cox analysis was carried out using the “survival” package (v 3.5-3), retaining genes with *P* < 0.05 and HR ≠ 1. These genes were subsequently subjected to proportional hazards assumption testing, and only those meeting the assumption (*P* > 0.05) were defined as candidate prognostic genes. LASSO regression was then applied using the “glmnet” package (v 4.1-4), with the optimal model determined by 5-fold cross-validation. Genes selected in this final model were designated as prognostic genes.

### 2.5 Real-time quantitative PCR (RT-qPCR)

Human-derived PCa cell lines LNCaP and normal prostate epithelial cell lines RWPE-1 were purchased from Cell Bank of Chinese Academy of Sciences (Shanghai, China). All lines were cultured in Dulbecco’s Modified Eagle Medium (Gibco, Thermo Fisher Scientific, Inc.) supplemented with 10% fetal bovine serum and 1% penicillin-streptomycin (Thermo Fisher Scientific, Inc.), then nurtured in 5% CO2 at 37 °C. All cells used in the experiment were maintained at 3–5 generations after recovery. Total RNA was extracted from cells with TRIzol reagent (Nordic Bioscience, Beijing, China) and reverse-transcribed into cDNA by reverse transcription kit (Promega, Madison, USA). The primer sequences of the specific genes (KIF4A, TPX2, and AURKB) are listed in Table 1. Reverse transcription was performed using the PrimeScript RT reagent Kit with gDNA Eraser (TaKaRa) according to the manufacturer’s instructions. Briefly, a genomic DNA (gDNA) removal mixture was prepared containing 1 μg of total RNA, 2 μL of 5× gDNA Eraser Buffer, 1 μL of gDNA Eraser, and nuclease-free water to a final volume of 10 μL. The reaction was incubated at 42 °C for 2 min. Subsequently, 4 μL of 5× PrimeScript Buffer, 1 μL of RT Primer Mix, 1 μL of PrimeScript RT Enzyme Mix I, and 4 μL of nuclease-free water were added, bringing the total volume to 20 μL. The reaction was then incubated at 37 °C for 15 min, followed by enzyme inactivation at 85 °C for 5 s. The synthesized cDNA was stored at −20 °C until use. Quantitative real-time PCR (qPCR) was conducted on a 7,500 fast real-time PCR system (Applied Biosystems, Foster City, CA, USA) with SYBR Green PCR Master Mix Kit (Nordic Bioscience, Beijing, China). Each 20 μL reaction contained 10 μL of SYBR Premix Ex Taq II, 0.8 μL each of forward and reverse primers (10 μM), 2 μL of cDNA template, and 6.4 μL of sterile water. The thermal cycling protocol consisted of an initial denaturation at 95 °C for 30 s, followed by 40 cycles of denaturation at 95 °C for 5 s and annealing/extension at 60 °C for 30 s, with fluorescence acquisition at this step. A melt curve analysis was performed immediately after amplification to confirm the specificity of the PCR products by heating from 65 °C to 95 °C in increments of 0.5 °C, with a 5-s hold at each step, while continuously monitoring fluorescence. The relative mRNA amounts were determined using 2^−ΔΔCT^ calculations with GAPDH as the control reference.

**TABLE 1:** Primer sequences for RT-qPCR experiment.

Genes	Accession number	Size	Forward (5‘-3’)	Reverse (5‘-3’)
KIF4A	NM_012310	189	AGCTTCTTTAATCCCGTCTGTG	GGCCAGAGCCCGTTTCTTT
TPX2	NM_012112	144	TCCTGCCCGAGTGACTAAGG	CTGTTAGGGGTTCGTTTATGGAA
AURKB	NM_004217	94	CAGTGGGACACCCGACATC	GTACACGTTTCCAAACTTGCC
GAPDH	NM_002046	116	ACATCAAGAAGGTG GTGAAGCAG	AAAGGTGGAGGAGTGGGTGTC

### 2.6 Immunohistochemistry (IHC) staining

IHC staining was employed to assess DEGs expression in PCa versus adjacent non-tumor tissues of 5 samples. Ethical approval for the study was granted by the First Affiliated Hospital of Guizhou University of Traditional Chinese Medicine (Number: KS2025012), and written informed consent was obtained from all participants. The diagnosis of PCa was established based on the criteria set by the NCCN guidelines (Schaeffer et al., 2023). We referred to the previous study (Yang et al., 2020) protocol to perform IHC staining. In brief, following antigen retrieval and blocking, tissue sections were incubated overnight at 4 °C with the following primary antibodies: anti-KIF4A (Proteintech, Cat No. 14344-1-AP, 1:100 dilution), anti-TPX2 (Servicebio, Cat No. GB111226, 1:400 dilution), and anti-AURKB (AiFang, Cat No. AFRM0124, 1:100 dilution). On the next day, the sections were incubated with an HRP-Polymer anti-rabbit secondary antibody kit (AiFang, Cat No. AFIHC003) at room temperature for 50 min. Color development was performed using a DAB chromogen substrate (20×) (AiFang, Cat# AFIHC004) for approximately 5 min. Subsequently, the nuclei were counterstained with hematoxylin, and the sections were mounted with neutral balsam (AiFang, Cat No. AFIHC043). Images were acquired using a light microscope (Olympus, Japan) at 200× magnification. Scoring was based on the H-score system, and statistical analysis employed Student’s t-test (mean ± SD).

### 2.7 Western blotting (WB) analysis

PCa and adjacent non-tumor tissues were homogenized in ice-cold RIPA lysis buffer supplemented with protease inhibitors (tissue:buffer ratio = 1:10, w/v). The homogenates were centrifuged at 10,000 × *g* for 10 min at 4 °C to collect the supernatants, which were subsequently stored at −20 °C until use. Protein concentrations were determined using a bicinchoninic acid (BCA) assay kit (Solarbio, Beijing, China). For immunoblotting, equal amounts of protein (50 μg) were denatured in SDS sample buffer, resolved by SDS-PAGE (80 V for 25 min, followed by 120 V for 50 min), and then transferred onto PVDF membranes (Millipore, MA, USA) at 200 mA for 2 h. After blocking with blocking buffer (NCM Bio, Cat No. P30500) for 10 min at room temperature, the membranes were incubated overnight at 4 °C with a primary antibody against butyryl-histone H3 (Lys27) (rabbit polyclonal, PTM Bio, Cat No. PTM-315, 1:1,000 dilution). Following five washes (5 min each) with TBST (TBS containing 0.1% Tween-20), the membranes were probed with an HRP-conjugated goat anti-rabbit secondary antibody (ZSGBBIO, Cat No. ZB-2301, 1:25,000 dilution) for 1 h at room temperature with gentle shaking. Protein bands were visualized using a Bio-Rad detection system (Quantity One, USA), and band intensities were quantified by densitometry with the same software. β-actin (Zen Bio, Cat No. R380624, 1:10,000 dilution) served as the loading control for normalization.

### 2.8 Construction and validation of the prognostic model

A risk model was constructed based on the identified prognostic genes. Using the optimal cut-off value of the risk score, risk score = (β_KIF4A × Expr_KIF4A) + (β_TPX2 × Expr_TPX2) + (β_AURKB × Expr_AURKB), where β is the coefficient obtained from LASSO-Cox regression and Expr is the gene expression value standardized by Z-score. 408 PCa samples with complete survival data from the TCGA-PRAD training cohort were stratified into high-risk and low-risk groups. To evaluate the model performance, Kaplan–Meier (K-M) survival curves were generated using the “survminer” package (v 0.4.9), and survival differences between the two groups were assessed by the log-rank test (*P* < 0.05). Time-dependent receiver operating characteristic (ROC) curves at 1, 3, and 5 years were generated using the “survivalROC” package (v 1.0.3.1). The model’s discriminatory power was assessed by calculating the area under the curve (AUC), which reflects the model’s ability to discriminate at different follow-up time points. Risk score distribution, survival status plots, and a heatmap of prognostic gene expression were visualized using the “survminer” and “pheatmap” (v 1.0.12) packages to illustrate disparities between risk groups. Finally, to assess the robustness and generalizability of the model, it was further validated in the validation cohort.

### 2.9 Clinical variables analysis and nomogram construction

To further evaluate the clinical utility of the risk model, differences in prognostic gene expression and risk scores across clinical variables (e.g., age, race) were analyzed using Wilcoxon rank-sum tests in 386 PCa samples from the TCGA-PRAD with complete clinical data. Independent prognostic factors were subjected to proportional hazards assumption testing, followed by multivariable Cox regression analysis (*P* < 0.05, HR ≠ 1). A nomogram was developed using the “rms” package (v 6.5.0) (Sachs, 2017) based on the independent factors. Its predictive performance was validated through 1-, 3-, and 5-year calibration curves, time-dependent ROC analysis (AUC > 0.7), and decision curve analysis (DCA) using the “ggDCA” package (v 1.6) (Chen Y. et al., 2025) to assess clinical utility.

### 2.10 Immune infiltration analysis and tumor mutation burden (TMB)

To explore the tumor immune microenvironment in PCa, the infiltration levels of 28 immune cell types in the high- and low-risk groups were quantified using single-sample gene set enrichment analysis (ssGSEA) based on the TCGA-PRAD training cohort (samples with *P. adj* > 0.05 were excluded). Differential immune infiltration between the two risk groups was assessed using the Wilcoxon rank-sum test. Spearman correlation analysis was performed to evaluate both the interrelationships among differentially infiltrated immune cells and the associations between prognostic genes and these immune cells (|cor| > 0.3 and *P. adj* < 0.05), using the cor () function in R (v 4.2.2). To investigate the TMB landscape, somatic mutation data were obtained from the TCGA-PRAD cohort. The “maftools” package (v 2.14.0) was employed to analyze mutational profiles and visualize the top 20 most frequently mutated genes via waterfall plots in each risk group.

### 2.11 Gene set enrichment analysis (GSEA) and drug sensitivity analysis

The reference gene set (c2.cp.kegg.v7.4.symbols.gmt) was obtained from the Molecular Signatures Database (http://www.gseamsigdb). GSEA was performed using the “clusterProfiler” package (v 4.7.1.003) to identify KEGG pathways significantly enriched in the high- and low-risk groups within the training cohort, with significance thresholds set at |NES| > 1 and *P. adj* < 0.05. To investigate the association between drug sensitivity and the risk model, drug response data were retrieved from the Genomics of Drug Sensitivity in Cancer (https://www.cancerrxgene.org/) database. Compounds exhibiting a significant correlation with risk scores (|cor| > 0.3 and *P. adj* < 0.05) were selected. Differences in the estimated half-maximal inhibitory concentration (IC_50_) values between the high- and low-risk groups were assessed using the Wilcoxon test.

### 2.12 Network of molecular regulation

To elucidate the molecular regulatory mechanisms of the prognostic genes, multiMiR package (v 1.20.0) (Ru et al., 2014) was applied to predict miRNAs. lncRNAs interacting with the identified miRNAs were predicted via the Encyclopedia of RNA Interactomes (starbase) with a threshold of clipExpNum >10. A transcription factors (TFs) regulatory network targeting the prognostic genes was constructed using NetworkAnalyst, and the integrated TFs–miRNA–gene network was visualized with Cytoscape (v 3.9.1).

### 2.13 Drug prediction and molecular docking

To explore potential therapeutic methods for PCa, the Comparative Toxicogenomics Database (https://ctdbase.org/) was queried to identify active compounds targeting the prognostic genes, with a selection threshold of Interaction. Count >4. The three-dimensional (3D) structure of the potential target protein encoded by the prognostic genes was retrieved from the Protein Data Bank (https://www.rcsb.org/), while the 3D conformation of the selected compound was downloaded from the PubChem database (https://pubchem.ncbi.nlm.nih.gov/). Molecular docking simulations was performed using the CB-Dock platform (https://cadd.labshare.cn/cb-dock2/php/index.php), with a binding energy threshold of <-1.2 kcal/mol indicating favorable binding activity. The docking pose with the lowest binding energy was selected for visual representation.

### 2.14 Statistical analysis

Bioinformatics analyses were executed utilizing the R software (v 4.2.2). Comparative analysis of data from different groups was carried out using the Wilcoxon test (*P* < 0.05).

### 2.15 Correlation analysis with BHD1

To investigate the Kbhb-specific association of the prognostic genes, we evaluated their correlation with BHD1 (β-hydroxybutyrate dehydrogenase), a key enzyme in β-hydroxybutyrate synthesis. Gene expression data from the TCGA-PRAD cohort were used. Spearman correlation analysis was performed to calculate correlation coefficients (cor) and adjusted *P*-values (*P. adj*), with significance thresholds set at |cor| > 0.3 and *P.adj* < 0.05 (Buck et al., 2026).

## 3 Results

### 3.1 Identification of 19 Kbhb-related candidate genes

**FIGURE 2:**
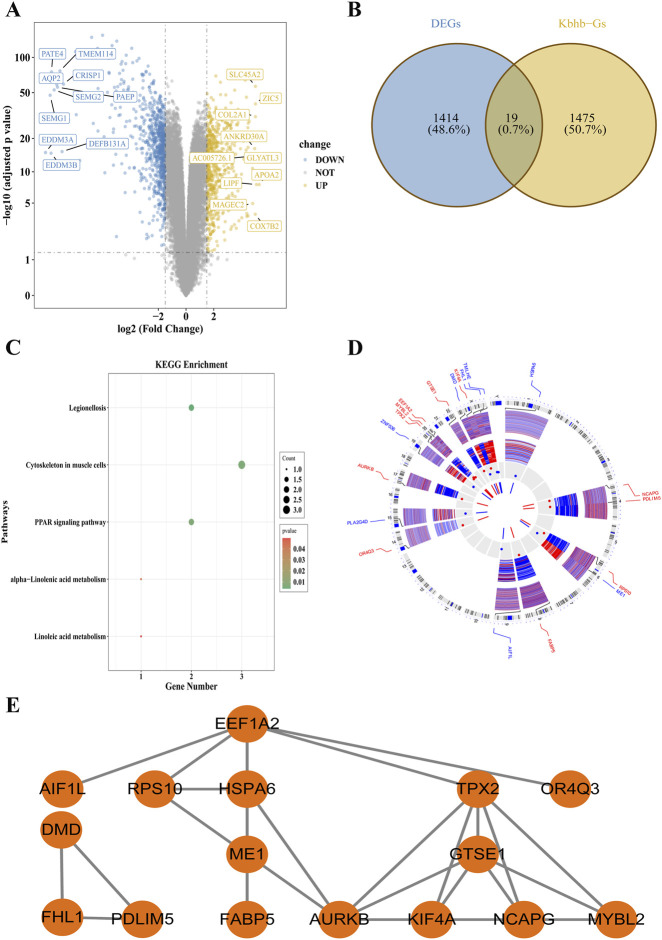
Identification of 19 candidate genes associated with Kbhb in PCa. **(A)** Volcano plot of DEGs between PCa and normal tissues. Red and blue dots represent significantly upregulated and downregulated genes (*P. adj* < 0.05, |log_2_FC| > 1.5), respectively. The top 10 up- and downregulated genes are labeled. **(B)** Venn diagram showing the intersection of 1,433 DEGs and 1,494 known Kbhb genes, yielding 19 candidate genes. **(C)** KEGG pathway enrichment analysis of the 19 candidate genes. **(D)** Chromosomal locations of the 19 candidate genes. Red and blue bars indicate up- and downregulation in PCa, respectively. **(E)** PPI network of the candidate genes (3 isolated proteins were observed in the analysis).

From the TCGA-PRAD training cohort, 1,433 DEGs were identified. Compared with the normal group, the PRAD group exhibited 624 upregulated and 809 downregulated genes (Figure 2A). By intersecting the 1,433 DEGs with 1,494 Kbhb genes, 19 candidate genes were obtained (Figure 2B; Supplementary Table S2), and many of these candidate genes were overexpressed in the PRAD group (Supplementary Figure S1). Functional enrichment analysis revealed that these 19 candidate genes were enriched in five pathways: Legionellosis, Cytoskeleton in muscle cells, PPAR signaling pathway, alpha-Linolenic acid metabolism, and Linoleic acid metabolism (Figure 2C; Supplementary Table S3). Chromosomal localization analysis indicated that TMLHE, FHL1, KIF4A, and DMD are located on the X chromosome, with KIF4A being upregulated and the others downregulated. ME1 and RPS10 are located on chromosome 6, among which RPS10 was upregulated and ME1 was downregulated (Figure 2D). PPI network analysis revealed a co-expression interaction between EEF1A2 and RPS10 (Figure 2E), suggesting that they may cooperatively participate in relevant molecular regulatory processes.

### 3.2 Prognostic gene-based risk model for PCa in the training cohort

**FIGURE 3:**
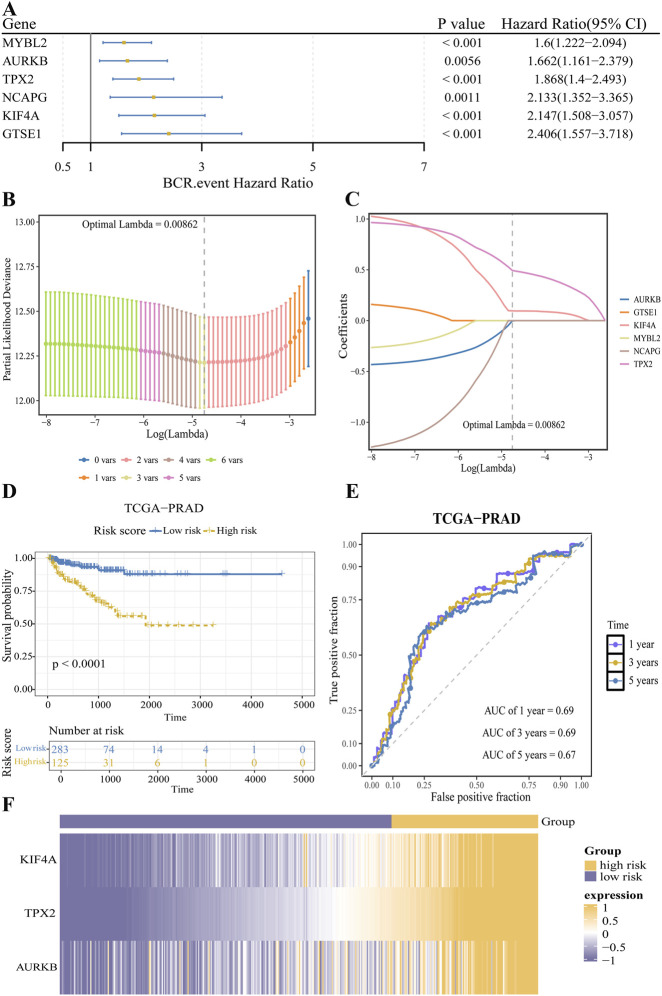
Construction of the prognostic gene-based risk model for PCa in the training cohort. **(A)** Univariate Cox regression analysis identified six genes (GTSE1, KIF4A, NCAPG, AURKB, MYBL2, TPX2) significantly associated with overall survival (OS). **(B)** LASSO regression analysis for variable selection. The vertical dashed line indicates the optimal value of the penalty parameter (λ) determined by 5-fold cross-validation. **(C)** Coefficient profile plot of the six genes from the LASSO regression. **(D)** K-M survival curves comparing the overall survival of patients in the high-risk and low-risk groups (*P* < 0.05, log-rank test). **(E)** Time-dependent ROC curves showing the discriminatory ability of the risk score for 1-, 3-, and 5-year OS. **(F)** Heatmap displaying the expression levels of the three final prognostic genes (KIF4A, TPX2, AURKB) in the high-risk and low-risk groups.

Through univariate Cox regression and PH test in the TCGA-PRAD training cohort, 6 prognostic genes: GTSE1, KIF4A, NCAPG, AURKB, MYBL2, and TPX2, were identified from the initial set of 19 candidates (Figure 3A). To refine the model, LASSO regression was performed, which retained three genes (KIF4A, TPX2, and AURKB) at a lambda value of 0.00862 (Figure 3B). A risk model was subsequently constructed using the following coefficients: KIF4A (0.096), TPX2 (0.493), and AURKB (−0.003) (Figure 3C). Based on an optimal risk score cutoff of 1.104, the 408 PCa patients with available survival data were categorized into high-risk (n = 125) and low-risk (n = 283) groups. K-M analysis showed that the high-risk group had a significantly lower survival probability than the low-risk group (Figure 3D). Time-dependent ROC analysis demonstrated AUC values of 0.69, 0.69, and 0.67 for 1-, 3-, and 5-year survival, respectively, indicating relative discriminatory ability of the risk model (Figure 3E). Moreover, the heatmap revealed elevated expression levels of KIF4A, TPX2, and AURKB (Supplementary Figure S2) in the high-risk group (Figure 3F; Supplementary Tables S1, S2).

### 3.3 Validation of the risk model and molecular expression in the validation cohort

**FIGURE 4:**
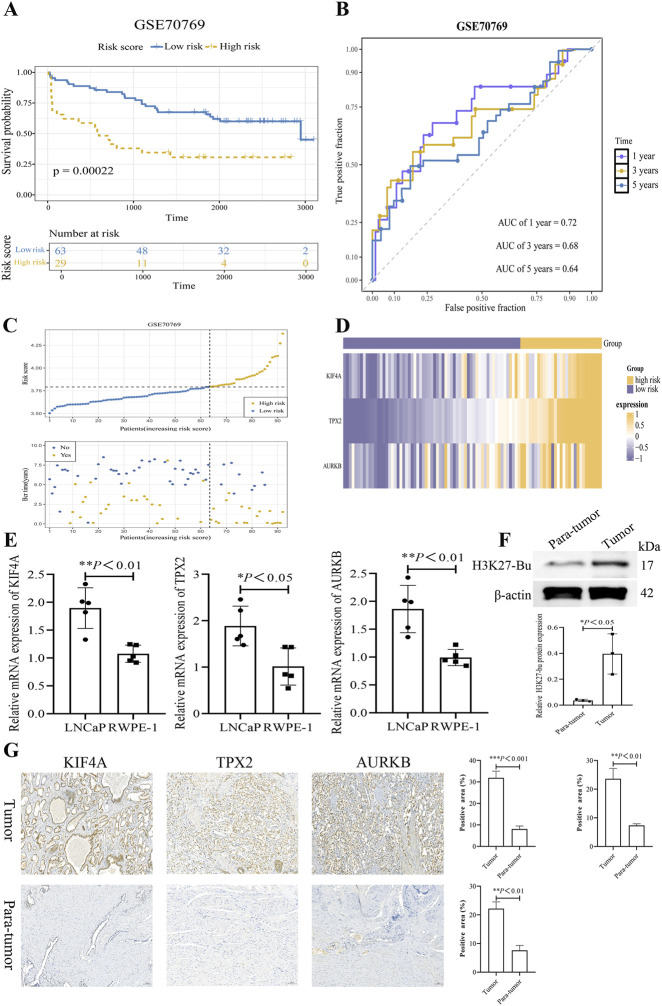
Validation of the prognostic gene-based risk model for PCa. **(A)** K-M survival analysis in the validation cohort, showing significantly worse overall survival for patients in the high-risk group (*P* < 0.05). **(B)** Time-dependent ROC curves at 1, 3, and 5 years in the validation cohort, with area under the curve (AUC) values indicating the model’s discriminatory ability. **(C)** Distribution of risk scores and patient survival status. The scatter plot shows an increasing number of death events (red dots) with higher risk scores. **(D)** Heatmap displaying the expression levels of KIF4A, TPX2, and AURKB across the risk groups in the validation cohort, confirming their significant upregulation in the high-risk group. **(E)** Relative mRNA expression levels of KIF4A, TPX2, and AURKB in the prostate cancer cell line LNCaP compared to the normal prostate epithelial cell line RWPE-1, as determined by RT-qPCR (***P < 0.01, **P < 0.01, *P < 0.05). **(F)** Protein expression levels of H3K27-Bu in PCa tissues compared to adjacent non-tumor tissues. **(G)** IHC images showing stronger positive staining for KIF4A, TPX2, and AURKB in PCa tissues compared to adjacent non-tumor tissues.

In the validation cohort, 92 PCa patients with complete survival data were stratified into a high-risk group (n = 29) and a low-risk group (n = 63) based on the optimal cutoff value (3.791719) of the risk score. The K-M survival curves showed a significantly lower survival probability in the high-risk group compared to the low-risk group (Figure 4A). AUC values for 1, 3, and 5 years were 0.72, 0.68, and 0.64, respectively, indicating stable and reliable discriminatory ability of the prognostic model (Figure 4B). Analysis of the risk curve and prognostic status plot suggested that as the risk score increased, the case of biochemical recurrence (BCR) also rose (Figure 4C). Furthermore, the heatmap revealed that the expression levels of KIF4A, TPX2, and AURKB were markedly elevated in the high-risk group (Figure 4D). Subsequent experimental validation by RT-qPCR confirmed that the expression levels of KIF4A (fold change = 1.8, *P < 0.05), TPX2 (fold change = 1.8, *P* < 0.01), and AURKB (fold change = 1.8, *P* < 0.01) were significantly upregulated in LNCaP compared with RWPE-1 cells, indicating consistent albeit modest changes that remain statistically robust (Figure 4E). Although fold changes are below 2.0, their reproducibility across assays supports their utility as biomarkers, this stable expression variation holds significant biological implications in clinical samples.

Then, we measured the butyrylation level of the H3K27 (H3K27-Bu) protein. Results indicated the butyrylation level was significantly upregulated in cancer tissues (*P* < 0.05, Figure 4F). IHC staining further identified that KIF4A, TPX2, and AURKB exhibited a substantially higher positive rate in cancer tissues relative to adjacent tissues (Figure 4G). These results establish KIF4A, TPX2, and AURKB as stable and reliable biomarkers for PCa progression and prognosis. Although the loading control β-tubulin exhibited consistent expression across samples, the quantitative densitometry analysis (normalized to β-tubulin) confirmed a specific increase in H3K27-Bu, indicating that the observed upregulation is relative and statistically robust. This suggests that Kbhb modification at H3K27 is selectively altered in PCa, independent of global protein changes.

### 3.4 Construction and validation of nomogram based on the risk score and prognostic genes

**FIGURE 5:**
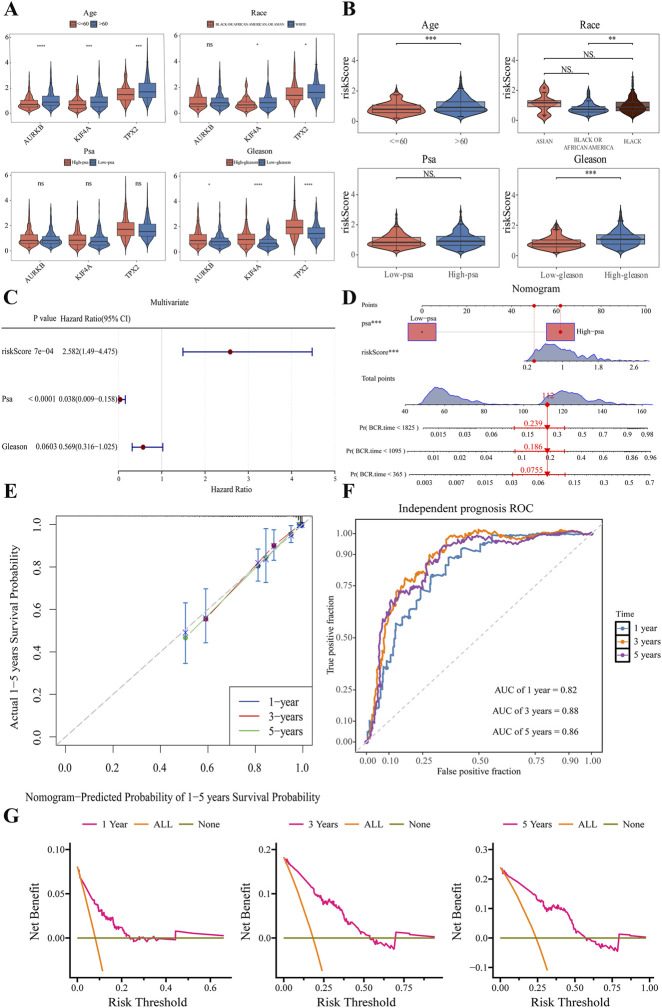
Nomogram construction and validation based on the risk score and prognostic genes. **(A)** Violin plot of KIF4A, TPX2, AURKB expression in clinical subgroups (age, race, and Gleason score). **(B)** Violin plot of risk score in clinical indices (age, race, and Gleason score). **(C)** Multivariate Cox regression analysis for independent prognostic factors. **(D)** Nomogram based on risk score; **(E)** Calibration curves for 1-, 3-, and 5-year survival probability. **(F)** ROC curve analysis of 1-, 3-, and 5-year prognosis. **(G)** DCA of nomogram for 1, 3, and 5 years.

Analysis of clinical variables revealed that the expression levels of KIF4A, TPX2, and AURKB exhibited significant disparities across different clinical variables stratified by age, race and Gleason score (Figure 5A). Furthermore, the risk score also showed significant differences among these clinical variables (Figure 5B). Univariate Cox regression analysis identified that the risk score, PSA, and Gleason score were significantly associated with patient prognosis (Supplementary Figure S3). Multivariate Cox regression analysis confirmed that both the risk score and PSA remained significantly associated with prognosis and were thus identified as independent prognostic factors (HR ≠ 1, *P* < 0.05) (Figure 5C). Based on these independent prognostic factors, a nomogram was constructed to predict the 1-, 3-, and 5-year survival probabilities of patients. The nomogram illustrates that a higher risk score corresponds to an increased BCR of PCa. Specifically, a total score reaching 112 points suggests a high BCR of Pca (Figure 5D). The calibration curves for the 1-, 3-, and 5-year predictions demonstrated high concordance with the reference line, indicating excellent consistency between the predicted probabilities and actual outcomes (Figure 5E). ROC curve analysis yielded AUC values of 0.82, 0.88, and 0.86 for predicting 1-, 3-, and 5-year survival, respectively, suggesting relative discriminatory ability of the nomogram (Figure 5F). Additionally, DCA revealed that the net benefit rates of the nomogram for 1, 3, and 5 years were substantially higher than both the “treat all” and “treat none” strategies across a wide range of threshold probabilities (Figure 5G). Those results indicate that the nomogram provides superior clinical utility and net benefit.

### 3.5 Correlation between prognostic genes and BHD1

**FIGURE 6:**
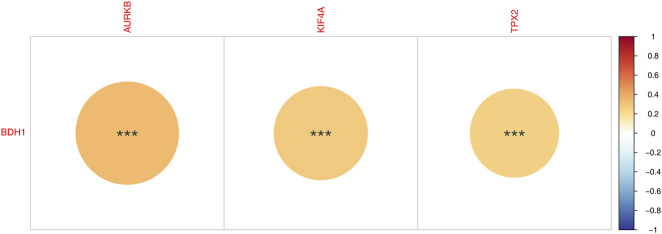
Correlation between KIF4A, TPX2, AURKB and BHD1.

To validate the Kbhb-specific relevance of the prognostic genes, we assessed their correlation with BHD1 expression. Spearman analysis revealed significant positive correlations: KIF4A and BHD1 (cor = 0.30, *P. adj* < 0.001), TPX2 and BHD1 (cor = 0.30, *P. adj* = 0.001), and AURKB and BHD1 (cor = 0.35, *P. adj* = 0.001). These results indicate that KIF4A, TPX2, and AURKB are potentially direct or indirect targets of Kbhb modification, supporting a pathway-specific discovery beyond proliferation-related functions (Figure 6).

### 3.6 Significant correlation between prognostic genes and immune cell infiltration

**FIGURE 7:**
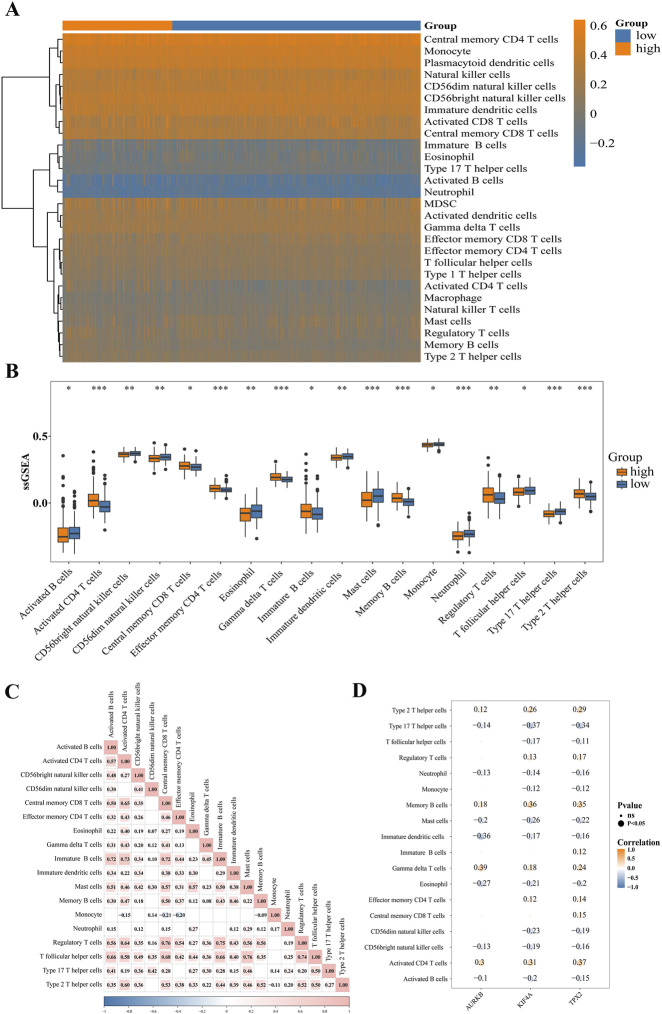
Immune microenvironment and correlation analysis between the high- and low-risk groups. **(A)** Hotmap of immune-infiltrating cells between the high- and low-risk groups. **(B)** ssGSEA of immune-infiltrating cells scores between the high- and low-risk groups (*P. adj* < 0.05). **(C)** Correlation analysis of immune-infiltrating cells. **(D)** Correlation analysis of immune-infiltrating cells with KIF4A, TPX2, AURKB (*P*. *adj* < 0.05).

To investigate the potential association between the aforementioned prognostic risk score and the tumor immune microenvironment (TIME), and to annotate the biological implications of the risk model, we performed an immune infiltration analysis. This analysis revealed a significantly higher abundance of central memory CD4^+^ T cells and monocytes in the low-risk group compared to the high-risk group (Figure 7A). Then, we identified 18 types of immune cells that exhibited significantly different infiltration levels between the high- and low-risk groups, including eosinophils, monocytes, and neutrophils (Figure 7B). Correlation analysis further showed that activated CD4^+^ T cells were significantly positively correlated with activated B cells (cor = 0.57, *P* = 2.43 × 10^−56^) but negatively correlated with monocytes (cor = −0.15, *P* = 0.005) (Figure 7C). In addition, we evaluated the associations between the prognostic genes and differentially infiltrated immune cells. A significant negative correlation was observed between Type 17 T helper cells and KIF4A expression (cor = −0.37, *P* = 2.64 × 10^−14^), whereas gamma delta T cells (γδ T cells) showed a significant positive correlation with AURKB expression (cor = 0.39, *P* = 3.12 × 10^−16^) (Figure 7D). These findings collectively indicated systemic remodeling of the immune microenvironment in PCa. The prognostic gene-immunocyte correlations suggested that KIF4A and AURKB might mediate immune-metabolic crosstalk, potentially linking Kbhb to PCa progression.

### 3.7 Differences of TMB, enriched pathways and drug sensitivity between high- and low-risk groups

**FIGURE 8:**
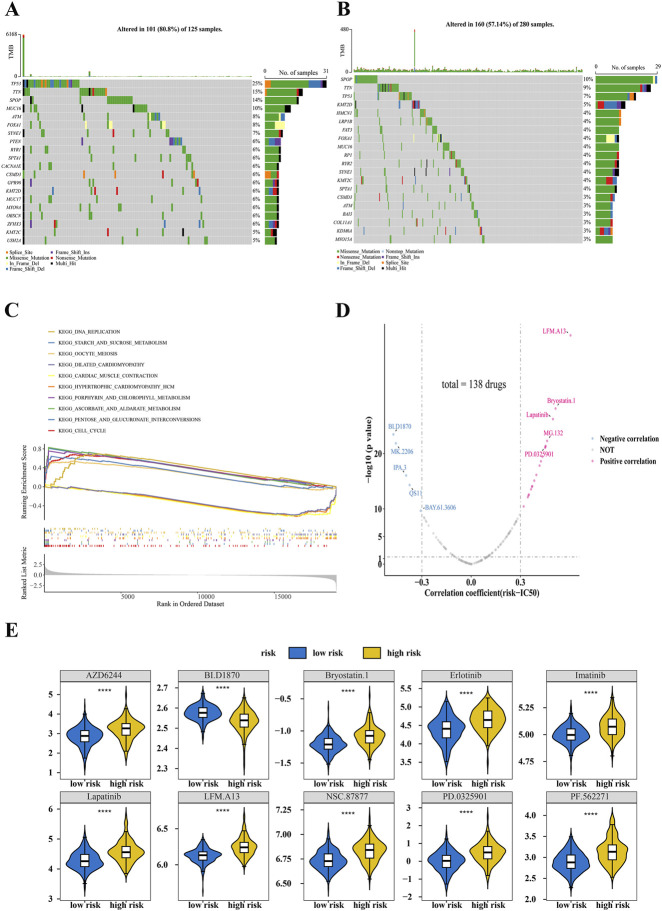
TMB, GSEA and drug sensitivity analysis between high- and low-risk groups. **(A)** Analysis of TMB in the high-risk group. **(B)** Analysis of TMB in the low-risk group. **(C)** GSEA between high- and low-risk groups (*P. adj* < 0.05, |NES| > 1). **(D)** Correlations between drug sensitivity and risk score. **(E)** Top 10 drugs with the most significant differences in IC_50_ between high- and low-risk groups (|cor|>0.3 *P. adj* < 0.05).

Analysis of TMB indicated that 101 out of 125 samples (80.8%) in the high-risk group harbored mutations. The top 3 mutated genes were TP53 (25%), TTN (15%), and SPOP (14%) (Figure 8A). In the low-risk group (n = 280), 160 samples (57.14%) exhibited mutations, with SPOP (10%), TTN (9%), and TP53 (7%) being the most mutated (Figure 8B). GSEA identified 50 significantly enriched pathways between the high- and low-risk groups. The top five most significantly enriched pathways were: cell cycle, pentose and glucuronate interconversions, ascorbate and aldarate metabolism and so on (Figure 8C).

Drug sensitivity analysis indicated that the IC_50_ values of 24 drugs were significantly correlated with the risk score, showing 19 positive and 5 negative correlations. Among them, Lapatinib demonstrated a significant positive correlation with the risk score (cor = 0.50, *P* = 5.78 × 10^−27^), whereas IPA.3 showed a significant negative correlation (cor = −0.40, *P* = 9.18 × 10^−17^) (Figure 8D). Furthermore, the IC_50_ values of these 24 drugs differed significantly between the high- and low-risk groups. The top 10 drugs with the most significant differences in IC_50_ were: AZD6244, BI.D1870, Brryostatin.1, Erlotinib, Imatinib, Lapatinib, LFM.A13, NSC.87877, PD.0325901, PF.562271 (Figure 8E; Supplementary Tables S4, S5).

### 3.8 Molecular regulatory network and drug targeting analysis of the prognostic genes

**FIGURE 9:**
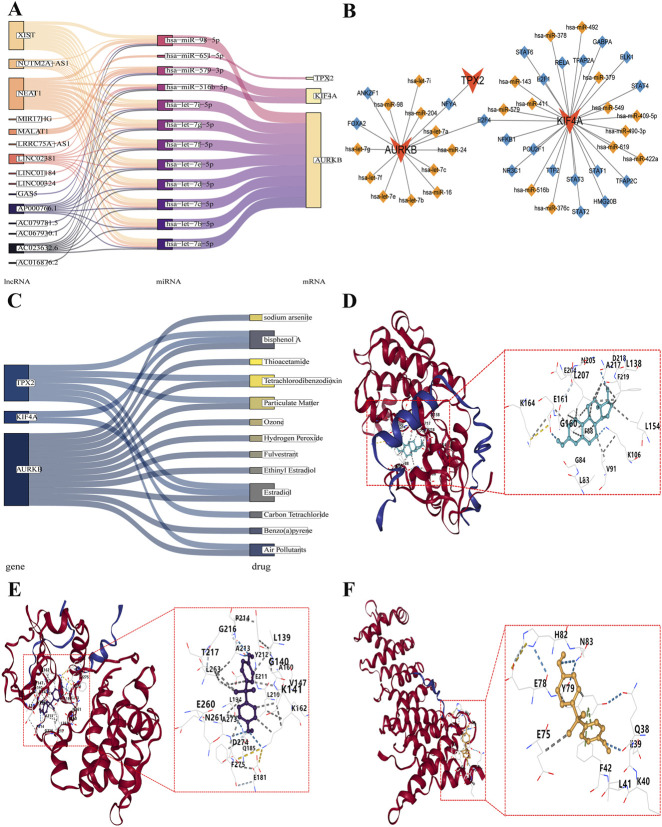
Molecular regulatory network and drug targeting analysis for KIF4A, TPX2, and AURKB. **(A)** Sankey diagram of lncRNA–miRNA–prognostic gene network. **(B)** Network of TFs–prognostic gene–miRNA. **(C)** Sankey diagram of prognostic gene-targeting drug. **(D)** Molecular docking of estradiol and AURKB. **(E)** Molecular docking of bisphenol A and TPX2. **(F)** Molecular docking of bisphenol A and KIF4A.

Based on the prognostic genes: KIF4A, TPX2, and AURKB, miRNA prediction was performed using the Miranda and Microcosm databases, which identified 45 and 55 miRNAs, respectively. The intersection of these miRNAs yielded 13 shared miRNAs, with specific miRNAs associated with each prognostic gene. AURKB was linked to 10 miRNAs (e.g., hsa-let-7e-5p, hsa-let-7a-5p), KIF4A to 2 miRNAs (hsa-miR-579-3p, hsa-miR-516b-5p), and TPX2 to 1 miRNA (hsa-miR-651-5p). Subsequently, using a threshold of clipExpNum >10, we identified 15 lncRNAs (including NEAT1, XIST, and LINC02381) that potentially regulate the above miRNAs, and constructed a lncRNA–miRNA–prognostic gene network. In this network, NEAT1 was predicted to sequester hsa-miR-516b-5p, then regulate KIF4A. Consistently, hsa-miR-516b-5p was shown to directly bind to the target sequence of KIF4A via complementary base pairing, leading to its suppression (Figure 9A). Furthermore, a total of 20 TFs were predicted, and a TFs–prognostic gene–miRNA regulatory network was established. Among them, POU2F1 and TTF2 were identified as potential regulators of KIF4A, while hsa-miR-549 was also implicated in the regulatory network modulating KIF4A (Figure 9B). Drug targeting analysis predicted 244 drugs for AURKB, 112 for KIF4A, and 184 for TPX2. Among these, 12 drugs target AURKB, 2 target KIF4A, and 6 target TPX2 with the criterion of interaction count >4. Bisphenol A and estradiol were identified as modulators capable of simultaneously targeting KIF4A, TPX2, and AURKB (Figure 9C). To validate the binding interactions between the biomarkers and their top-scoring ligands, molecular docking simulations were performed. The results showed that AURKB exhibited the strongest binding affinity with estradiol, achieving a binding energy of −9.8 kcal/mol, with key interactions at residues G160 and G84 (Figure 9D; Table 2). TPX2 showed a moderate binding affinity with bisphenol A (−7.8 kcal/mol), involving residue L194 (Figure 9E; Table 2). In contrast, the binding affinity between KIF4A and bisphenol A, with a binding energy of −6.5 kcal/mol and the key interaction at Y79 (Figure 9F; Table 2). Bisphenol A, which is associated with environmental exposure and endogenous signaling, indirectly suggests that core genes may regulate processes such as hormone signaling; the specific mechanisms remain to be elucidated. The binding energies observed in molecular docking (below −1.2 kcal/mol) suggest potential interactions between these compounds and the target proteins. While these computational results provide preliminary insights for hypothesis generation, the binding energies observed in molecular docking support the regulatory value of these compounds as modulators of KIF4A, TPX2 and AURKB activity in PCa treatment.

**TABLE 2:** Binding energy of estradiol, bisphenol A to the prognostic genes.

Target	Compounds	Binding affinity (kcal/mol)
AURKB	Estradiol	−9.8
TPX2	Bisphenol A	−7.8
KIF4A	Bisphenol A	−6.5

## 4 Discussion

PCa ranks among the most common malignancies of the male genitourinary system and poses a significant threat to men’s health. Notably, evidence has highlighted the critical role of epigenetic modifications in PCa progression. Among these, Kbhb has recently been identified as a novel post-translational modification that regulates gene expression and influences key biological processes such as tumor cell proliferation and metabolism (Chen C. et al., 2025; Qin et al., 2024; Zhu and Huang, 2025). Nevertheless, the expression patterns, prognostic value, and molecular regulatory mechanisms of Kbhb-related genes in PCa remain poorly characterized. In this study, we identified KIF4A, TPX2, and AURKB as key prognostic genes associated with Kbhb modification, highlighting their potential roles in the pathogenesis, diagnosis, and precision treatment of PCa. Based on the prognostic genes, we constructed a risk model that demonstrated robust predictive performance across both training and validation cohorts. Furthermore, we established both the risk score and PSA as independent prognostic factors in PCa. The nomogram incorporating these factors exhibited excellent discriminatory ability, along with superior clinical utility and net benefit as determined by DCA. Additionally, drug sensitivity analysis indicated significant correlations between the risk score and IC_50_ values for several compounds, including Lapatinib and IPA-3. Importantly, our analysis identified estradiol as a high-affinity ligand targeting AURKB, while bisphenol A showed potential for simultaneously targeting both TPX2 and KIF4A. These findings provide valuable evidence to support the development of precision treatment strategies and novel therapeutic agents for PCa.

This study identified kinesin family member 4A (KIF4A), targeting protein for *xenopus* kinesin-like protein 2 (TPX2), and aurora kinase B (AURKB) as key prognostic genes associated with Kbhb modification in PCa. KIF4A is a microtubule-dependent motor protein critical for chromosome segregation and spindle formation during cell division (Yang et al., 2020; Sachs, 2017; Chen Y. et al., 2025; Ru et al., 2014). Dysregulation of KIF4A has been increasingly implicated in oncogenesis. In hepatocellular carcinoma, KIF4A is transcriptionally activated by FOXM1, forming an oncogenic FOXM1-KIF4A axis that promotes tumor proliferation (Hu et al., 2019). In PCa, a previous study reported that KIF4A overexpression correlates with aggressive disease features and reduced recurrence-free survival, highlighting its potential as both a diagnostic marker and therapeutic target (Chen et al., 2020). In addition, upregulation of KIF4A correlates significantly with BCR and earlier occurrence of prostate-specific antigen (PSA) failure, as evidenced by survival analyses (Liu et al., 2025; Gao et al., 2018). This current study expands this understanding by identifying KIF4A as a Kbhb-related gene with prognostic relevance in PCa. We propose that Kbhb modification may enhance KIF4A’s microtubule-binding affinity and motor activity, thereby accelerating spindle assembly and chromosome segregation (Gluszek-Kustusz et al., 2023; Hannabuss et al., 2019). Furthermore, KIF4A was established as a risk factor in the risk model and demonstrated a negative correlation with tumor-infiltrating eosinophils (cor = −0.21). This suggests a dual mechanism whereby KIF4A promotes PCa progression—both by driving aberrant mitotic processes and by potentially suppressing anti-tumor immune responses.

TPX2 is a highly conserved microtubule-associated protein that localizes to the nucleus during interphase. It is essential for mitotic spindle assembly through its interaction with tubulin and activation of AURKA, and also participates in DNA damage repair via ATM kinase (Gao et al., 2018; Gluszek-Kustusz et al., 2023). Aberrant TPX2 expression has been closely associated with malignant phenotypes in various cancers. In ovarian cancer (OC), TPX2 promotes cancer cell proliferation and migration through regulation of Polo-like kinase 1 (PLK1) expression (Ma et al., 2018). While the role of TPX2 in PCa remains insufficiently established. This study provides the crucial evidence identifying TPX2 as a Kbhb-related prognostic gene in PCa, with its expression being regulated by Kbhb modification. We hypothesize that Kbhb modification may enhance TPX2’s interaction with AURKA through lysine residue modification, thereby promoting the formation of functional complexes that facilitate aberrant spindle assembly (He et al., 2022; Guo et al., 2019). Consistent with its mechanistic roles, our results identified TPX2 as a risk factor enriched significantly in the mitotic spindle assembly pathway, indicating its contribution to PCa progression through the induction of genomic instability driven by abnormal cell division. AURKB, a key aurora kinase family member, orchestrates chromosome segregation and cytokinesis via histone H3 phosphorylation (promoting chromatin condensation) and centromeric protein phosphorylation (maintaining centromere integrity) (Guo et al., 2019; Li J. et al., 2024; Li L. et al., 2024; Reichman et al., 2019; Jiang et al., 2024). The oncogenic role of AURKB has been extensively documented across various cancers (Li J. et al., 2024). In colorectal cancer (CRC), AURKB overexpression epigenetically activates cyclin E1 expression, thereby promoting tumor cell proliferation and growth (Li L. et al., 2024). In PCa, our study for the first time establishes AURKB as a Kbhb-related prognostic biomarker. Its expression level is significantly elevated in tumor tissues, which is consistent with previous findings of its high expression in cancers. We propose that Kbhb modification may enhance AURKB’s kinase activity and protein stability, consequently amplifying phosphorylation of its downstream substrates. Furthermore, our analysis revealed a significant positive correlation between AURKB expression and γδ T-cell infiltration (cor = 0.39), suggesting AURKB’s potential involvement in modulating the immune microenvironment to facilitate PCa progression.

Although previous studies indicate that β-hydroxybutyrate (βhb) via Kbhb modification can inhibit PCa cell proliferation, migration, and invasion [20], our study identifies KIF4A, TPX2, and AURKB as Kbhb-related genes that promote tumor progression. This apparent contradiction may stem from the context-dependent duality of Kbhb modification. Specifically, βhb might exert tumor-suppressive effects by globally modulating metabolic pathways or specific histone sites (e.g., H3K27), whereas in PCa, the dysregulation of key genes like KIF4A, TPX2, and AURKB—possibly through site-specific Kbhb modifications or indirect regulatory networks—could override these effects and drive oncogenic processes. Such dual roles are common in post-translational modifications, where the net outcome depends on cellular context, gene specificity, and interaction with other signaling pathways. Thus, our findings highlight the complexity of Kbhb-mediated regulation in PCa, suggesting that its role is not uniformly inhibitory but rather balanced by pro-tumorigenic mechanisms in specific subsets.

Subsequently, we developed a prognostic risk model based on the expression patterns of KIF4A, TPX2, and AURKB. Patients in the high-risk group exhibited significantly poorer BCR than those in the low-risk group, confirming the model’s strong predictive capability. Consistent performance in both training and validation cohorts further supported its robustness and generalizability, underscoring its potential for clinical application. By enabling precise risk stratification, the model offers a foundation for individualized therapeutic strategies aimed at improving patient outcomes.

We found significant differences in eosinophil, monocyte, and neutrophil infiltration between the high- and low-risk groups, reflecting distinct immune response capacities among patients in different risk groups. Eosinophils, recognized for anti-tumor properties, exert cytotoxic effects through the release of cytotoxic granules that directly eliminate tumor cells or modulate immune responses (Reichman et al., 2019). As essential components of the innate immune system, monocytes and neutrophils infiltrating the tumor microenvironment may be associated with inflammatory responses and immune escape mechanisms in cancer (Jiang et al., 2024; Dounousi et al., 2021). This suggests that the poor prognosis in high-risk patients may not only stem from the high proliferative capacity of cancer cells themselves, but also be associated with the immunosuppressive environment they create. We found a notably strong positive correlation between activated CD4^+^ T cells and activated B cells (cor = 0.57), suggesting coordinated anti-tumor activity. Activated CD4^+^ T cells provide critical stimulation to B cells through CD40 ligand (CD40L) secretion, driving their differentiation into plasma cells capable of producing tumor-specific antibodies (Wennhold et al., 2017). Conversely, B cells enhance CD4^+^ T cell responses through antigen presentation and enhanced cytokine secretion (Elizondo et al., 2017). This bidirectional interaction, forming “T-B cell immune synapses,” systematically amplifies anti-tumor immunity and provides a theoretical foundation for combination immunotherapy strategies (Yin et al., 2016). In contrast, a significant negative correlation was detected between activated CD4^+^ T cells and monocytes (cor = −0.15). Activated CD4^+^ T cells typically promote monocyte differentiation into pro-inflammatory M1-type macrophages through interferon-γ secretion, thereby enhancing anti-tumor immunity (Yoshimori et al., 2021). However, in the PCa microenvironment, tumor-derived immunosuppressive factors including interleukin-10 (IL-10) and transforming growth factor-β (TGF-β) skew monocyte differentiation toward immunosuppressive M2-type tumor-associated macrophages (TAMs) (Czaja, 2022). M2-TAMs create an immunosuppressive feedback loop through several mechanisms. They deplete L-arginine by secreting arginase-1, which impairs the proliferation and function of CD4^+^ T cells. At the same time, they engage programmed death-ligand 1 (PD-L1) receptors on T cells, directly inhibiting the activity of CD4^+^ T cells (Martinenaite et al., 2025). This immunosuppressive cascade not only dampens anti-tumor immunity but may also facilitate tumor progression and metastasis. These immunological findings collectively indicate systemic immune landscape remodeling in high-risk PCa patients, supporting the value of our prognostic model based on Kbhb-related features. Consequently, therapeutic interventions targeting this immunosuppressive feedback loop may represent promising strategies for PCa immunotherapy.

Drug sensitivity and targeting analysis showed a positive correlation between lapatinib and the risk score (cor = 0.50), suggesting potential drug resistance mechanisms in the high-risk group. As a dual inhibitor of epidermal growth factor receptor/human epidermal growth factor receptor 2 (EGFR/HER2), lapatinib’s efficacy relies on effective blockade of both signaling pathways (Tevaarwerk and Kolesar, 2009). However, in high-risk patients, aberrant activation of cell cycle pathways enriched by prognostic genes may form a cross-activation network with EGFR/HER2 signaling. On one hand, the PI3K-Akt pathway downstream of EGFR/HER2 could enhance proliferative signaling via cyclin phosphorylation, thereby attenuating lapatinib’s inhibitory effects (Mora Vidal et al., 2018; Pires et al., 2019). On the other hand, chromosomal instability commonly observed in high-risk groups may lead to EGFR/HER2 gene amplification or activating mutations, directly reducing drug-target binding efficiency. These findings imply that clinical application of lapatinib in high-risk patients necessitates genetic profiling to identify sensitive subtypes, or combination therapy with cell cycle inhibitors to overcome resistance (Hisamatsu et al., 2016).

In contrast, the significant negative correlation between IPA-3 (a specific PAK1 inhibitor) and the risk score (cor = −0.40) suggests a potential therapeutic alternative for high-risk patients (Shahinozzaman et al., 2020). IPA-3 exerts its effects through specific inhibition of PAK1, which is frequently overexpressed in high-risk groups due to upstream signaling hyperactivation and demonstrates functional synergy with prognostic genes including AURKB and TPX2-AURKB-mediated phosphorylation enhances PAK1 activity (Gerçeker et al., 2015), while TPX2-mediated spindle abnormalities rely on PAK1-dependent cytoskeletal regulation to maintain malignant phenotypes (Zhang et al., 2022). Therefore, IPA-3 may simultaneously suppress tumor proliferation and invasion through PAK1 inhibition, warranting further clinical validation. Furthermore, our study identified that estradiol exhibited the strongest binding affinity with AURKB. This interaction might inhibit spindle assembly checkpoint activation, leading to mitotic and cell cycle arrest (Lee et al., 2015) and ultimately promoting apoptosis in CRC cells. Additionally, we found that bisphenol A showed binding affinity with both TPX2 and KIF4A. This could be explained by bisphenol A-induced centriole overduplication and premature separation, resulting in multipolar spindle formation and disrupted proper localization of TPX2 to spindle microtubules, thereby compromising spindle polarity and eventually triggering apoptosis (Kim et al., 2019). Drug sensitivity analysis revealed differential responses to specific agents among PCa patients, facilitating more precise and effective personalized treatment strategies. Meanwhile, drug targeting analysis provides a scientific rationale for developing novel therapeutic agents and approaches for PCa.

Conclusively, this study has identified KIF4A, TPX2, and AURKB as Kbhb-related prognostic genes in PCa, and the constructed risk model exhibits substantial prognostic predictive value. However, several limitations of this study should be noted. Firstly, the specific biological functions of these prognostic genes, particularly their regulation by Kbhb modification, remain to be experimentally validated using techniques such as site-specific mutagenesis or co-immunoprecipitation. Secondly, the retrospective design and the sample size of the validation cohort, although informative, necessitate further prospective studies with larger, multi-center cohorts to confirm the clinical translatability and robustness of our risk model. Thirdly, the lack of *in vivo* validation (e.g., using animal models) for the oncogenic roles of KIF4A, TPX2, and AURKB, as well as the efficacy of the predicted compounds, represents a significant limitation. Forth, although Western blot analysis confirmed the upregulation of H3K27Bu, it failed to comprehensively analyze changes at other Kbhb sites (e.g., H3K9). While our bioinformatic analysis identified Kbhb-related prognostic genes (e.g., KIF4A, TPX2, AURKB), it did not specify the lysine β-hydroxybutyryl acyltransferase homologs responsible for these modifications. This is a limitation, as different acyltransferases (e.g., SIRT family members or KAT2A) may have distinct functions in PCa. Future research directions should therefore include: (1) functional experiments to delineate the causal relationship between Kbhb modification and the activity of the identified prognostic genes; (2) large-scale, prospective clinical trials to validate the prognostic accuracy of the model; (3) *in vivo* studies to assess the therapeutic potential of targeting the Kbhb-mitosis axis in PCa animal models; (4) Histone modification omics technology should be used to systematically identify the specific Kbhb modification profile in PCa; and (5) Future studies should validate specific enzymes using techniques like co-immunoprecipitation or histone modification omics.

## Data Availability

The datasets presented in this study can be found in online repositories. The names of the repository/repositories and accession number(s) can be found in the article/Supplementary Material.
