# Supplementary figures and images for: Prognostic biomarkers and regulatory mechanisms associated with lysine β-hydroxybutyrylation modification in prostate cancer
Source: Front Mol Biosci. 2026 Apr 10;13:1778536. doi: 10.3389/fmolb.2026.1778536 (PMC13105968; doi:10.3389/fmolb.2026.1778536)

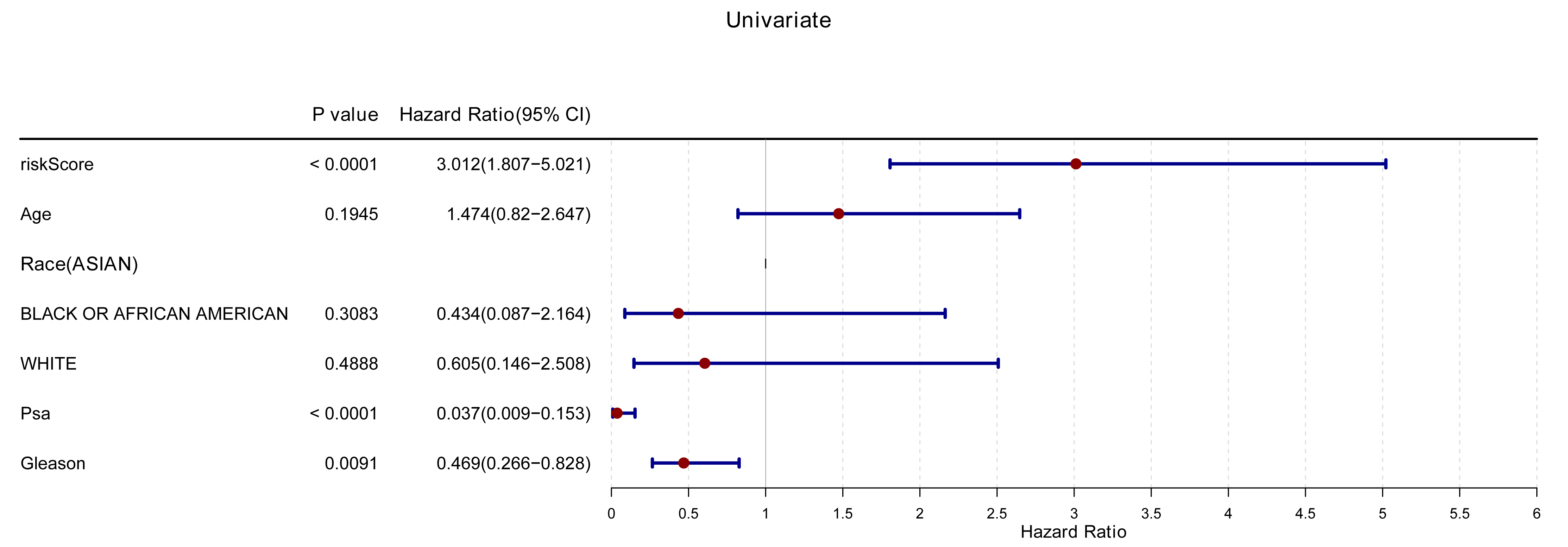

Supplement: Supplementary file 3 [file Image3.tif]

**R1**

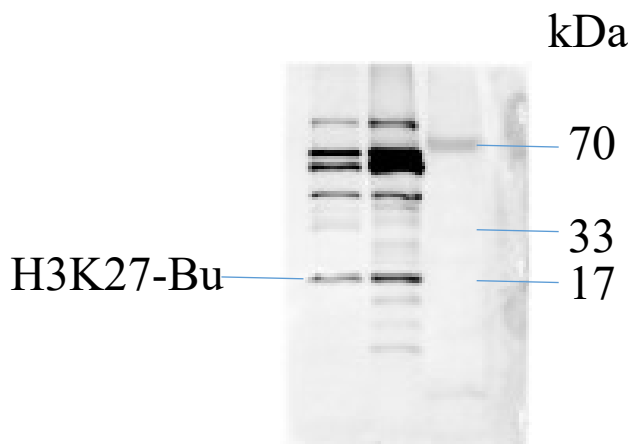

**R2**

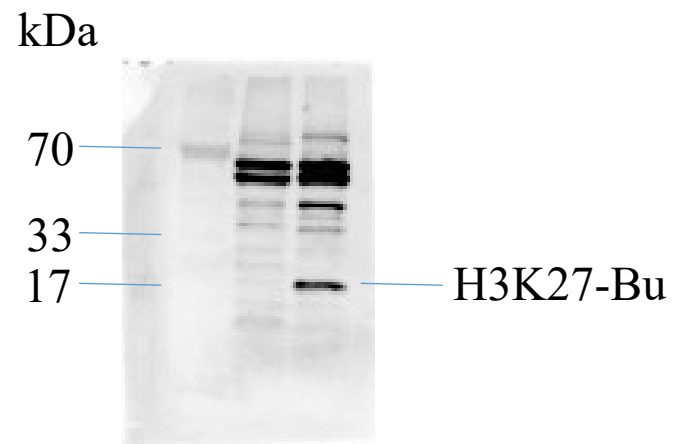

**R3**

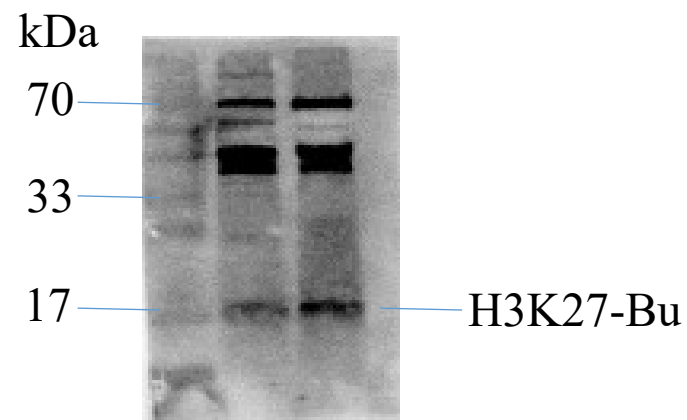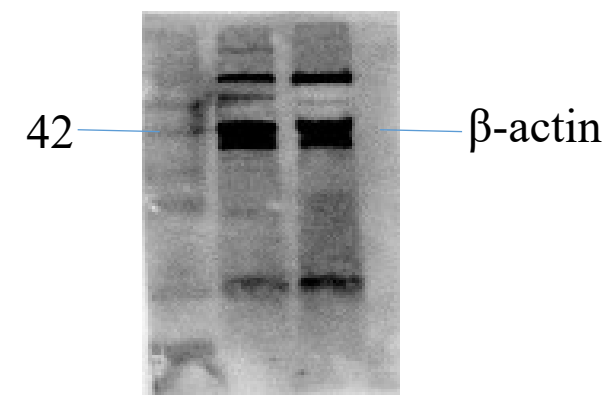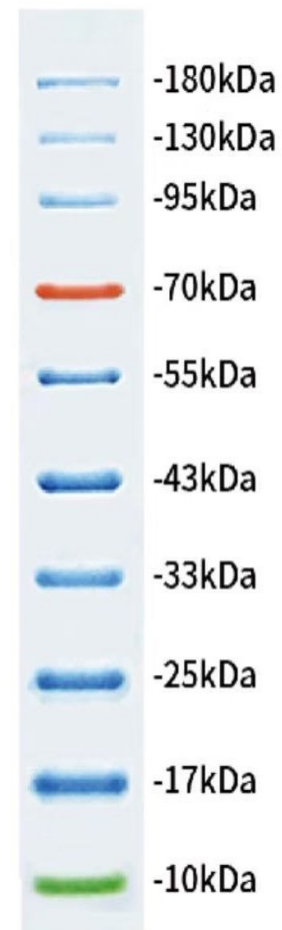

Supplement: Supplementary file 4 [file DataSheet1.pdf]

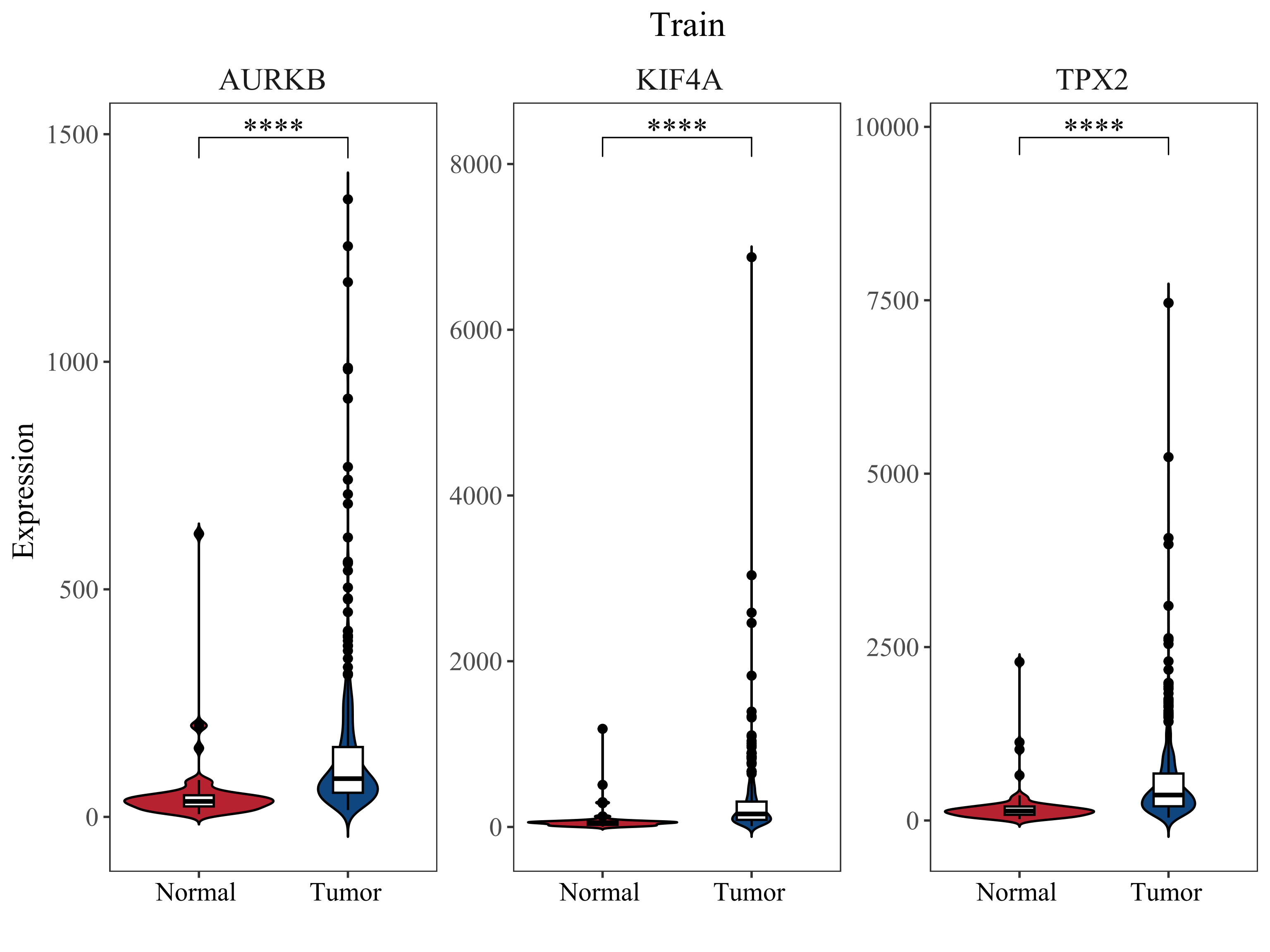

Supplement: Supplementary file 6 [file Image2.png]

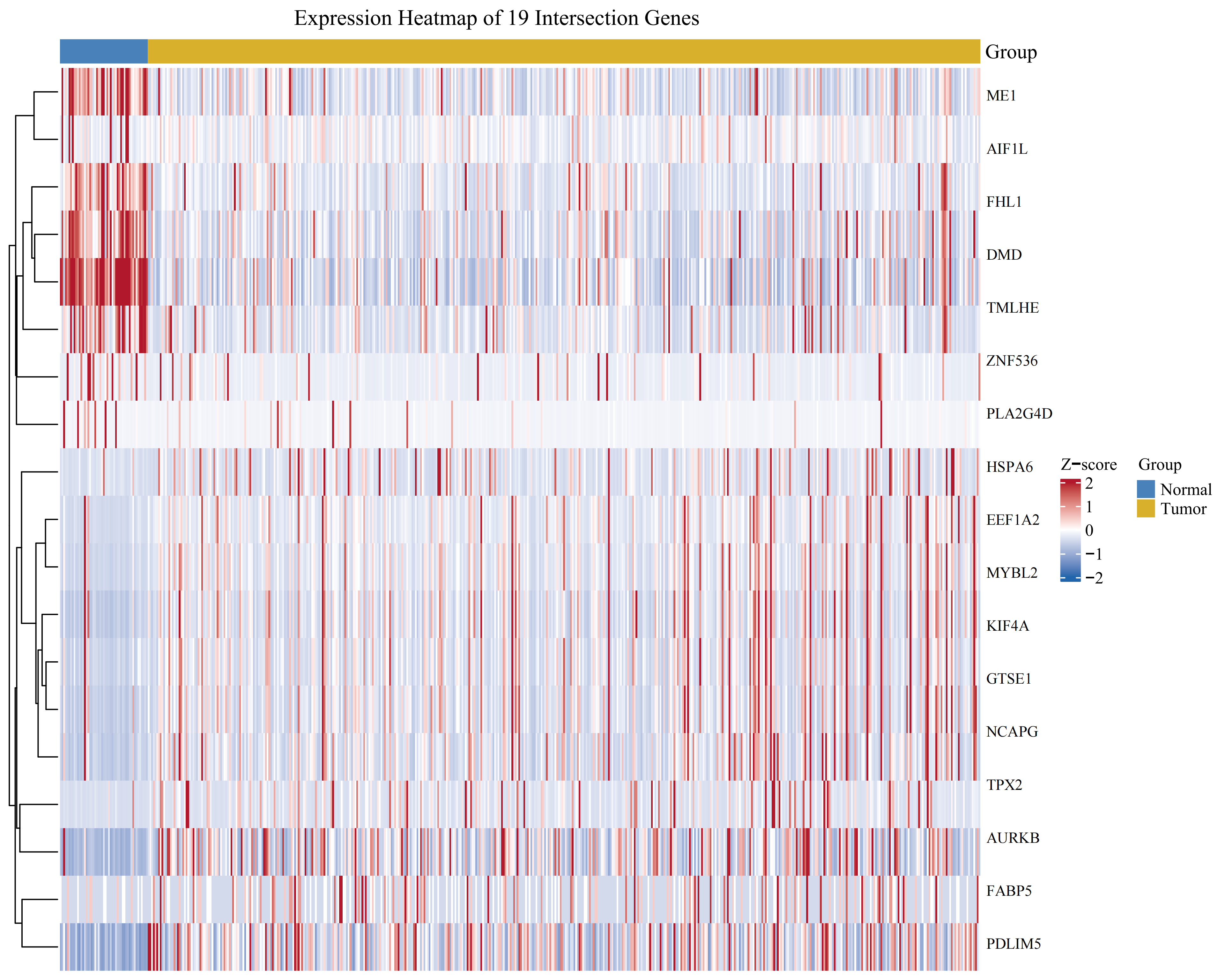

Supplement: Supplementary file 8 [file Image1.png]
